# Nurses and Voluntary Assisted Dying: How the Australian Capital Territory’s Law Could Change the Australian Regulatory Landscape

**DOI:** 10.1007/s11673-024-10370-y

**Published:** 2024-06-13

**Authors:** R. Jeanneret, S. Prince

**Affiliations:** 1 Medical School and T. C. Beirne School of Law, University of Queensland, 288 Herston Rd, Herston, QLD 4006 Australia; 2 Australian Centre for Health Law Research, Queensland University of Technology, 2 George Street, Brisbane, QLD 4000 Australia

**Keywords:** Voluntary assisted dying, Nurses, Nurse practitioners, Barriers to access, End-of-life care

## Abstract

On June 5, 2024, the Australian Capital Territory passed a law to permit voluntary assisted dying (“VAD”). The Australian Capital Territory became the first Australian jurisdiction to permit nurse practitioners to assess eligibility for VAD. Given evidence of access barriers to VAD in Australia, including difficulty finding a doctor willing to assist, the Australian Capital Territory’s approach should prompt consideration of whether the role of nurses in VAD should be expanded in other Australian jurisdictions. Drawing on lessons from Canada, which currently permits nurse practitioners to assess patient eligibility, we argue that the time has come for Australian jurisdictions to expand the role of nurses in VAD systems. This would be an important step in ensuring access to VAD for patients in practice. Attention, however, must also be paid to ensuring adequate remuneration of nurses (and doctors) if this goal of promoting access is to be achieved in practice.

## Introduction

Since 2017, all Australian states have passed laws permitting voluntary assisted dying (“VAD”). Victoria was the first state to pass a law with its VAD system commencing in June 2019, and New South Wales was the last, with its VAD system commencing in November 2023 (Waller et al. 2023). This means VAD is now a lawful end-of-life choice for most Australians. While there are differences between each state’s VAD framework, all states generally follow the approach taken in Victoria, resulting in the emergence of an “Australian model” for VAD that is “highly prescriptive” (Waller et al. 2023). To access VAD, each state’s law requires, among other things, the patient to make multiple requests for VAD and to have their eligibility assessed by two medical practitioners independently of each other. The patient must also decide on how the medication will be administered, which could include (in some states) administration by a nurse practitioner (“NP”) or registered nurse (“RN”).

The Australian Capital Territory (“ACT”) and Northern Territory (“NT”) were prohibited from legislating in relation to VAD as a result of a Commonwealth law passed in the 1990s. This law was overturned in 2022, which has enabled the ACT and NT to consider law reform (Waller et al. 2023). On June 5, 2024, the ACT passed the Voluntary Assisted Dying Bill 2023 (ACT) (“ACT Law”) which outlines a legislative framework to permit VAD.^1^ In some respects, the ACT Law follows the Australian model but in other respects it is unique. One aspect where it differs from the Australian model is the role of NPs. The ACT Law allows NPs to act in the role of a “coordinating practitioner” or “consulting practitioner” for a patient (i.e., NPs will be able to assess a patient’s eligibility for VAD). While some other Australian states allow NPs or RNs to administer VAD medications for patients who have been assessed as eligible, no state currently allows nurses to assess eligibility. In this regard, the ACT Law represents a significant departure from the Australian model.

The ACT’s approach to including NPs has stemmed from a concern about the small healthcare workforce in the ACT and the need to ensure access to VAD for patients in practice (Groves 2023). This concern resonates across Australian states. For example, a recent study in Victoria highlighted that finding a doctor to assess a patient’s eligibility for VAD was a key barrier to access (White et al. 2023). The ACT’s approach highlights an opportunity for other Australian states to consider an expanded role for nurses in VAD systems in light of emerging evidence of access barriers for patients (White et al. 2023), as well as concerns about workforce sustainability (Haining, et al. 2023a, b). Although the role for NPs in the ACT is unique in Australia, there is international precedent for NPs assessing patients’ eligibility for VAD. For example, NPs may assess and provide medical assistance in dying (“MAiD”) (as it is known locally) in Canada. In considering how an expanded role for NPs could operate, international experience may be useful for Australian states to consider.

This article first provides an overview of how nurses can currently be involved in VAD across Australian states and their expanded role under the ACT Law. It then examines NPs’ involvement in Canada’s MAiD system and highlights some ongoing challenges and considerations, such as remuneration, which may be relevant for Australia. Finally, this article considers what might be next for Australia in terms of a potentially expanded role for nurses in VAD frameworks.

## The Role of Nurses in Australian VAD Systems

The status of nurse involvement in VAD processes in Australia varies depending on the jurisdiction (Waller et al. 2023). Table 1 describes what “formal roles” (i.e., coordinating practitioner, consulting practitioner, or administering practitioner) nurses can act in during the VAD process. One commonality across the states and in the ACT Law is that only nurses who have undertaken legally mandated training are eligible to be administering practitioners (Waller et al. 2023). Table 1 also provides examples of how nurses may interact with VAD during their practice beyond acting in formal roles, such as supporting the general care of patients seeking VAD or initiating discussions to provide information about VAD. Nurses engaging in these non-formal roles are not generally required to have undertaken VAD or end-of-life law training.

**Table 1:** Status of nurse involvement in VAD process across Australian jurisdictions

**Jurisdiction**	**Formal Roles**		**Other Potential Interactions with VAD systems**					
	**Coordinating/Consulting Practitioner (i.e., assess eligibility)**	**Administering Practitioner (i.e., administer VAD medication)**	**Supporting general care of patient**	**Initiate discussion about VAD**	**Contact Person**	**Witness**		**Providing a determination about certain eligibility criteria**
						**Written request for VAD**	**Administration of VAD medication**	
Victoria	No	No	Yes (NPs and RNs)	No (can provide info on request)	Yes (NPs and RNs)	Yes (NPs and RNs) (if not directly involved in providing healthcare)	Yes (NPs and RNs) (must be independent of coordinating medical practitioner)	Yes (NPs and RNs)
*Voluntary Assisted Dying Act 2017* (Vic)								
Western Australia (‘WA’)	No	Yes (NPs)	Yes (NPs and RNs)	Yes (NPs)	Yes (NPs and RNs)	Yes (NPs and RNs)	Yes (NPs and RNs) (if not employed, or contracted by administering practitioner)	Yes (NPs and RNs)
*Voluntary Assisted Dying Act 2019* (WA)								
Tasmania	No	Yes (NPs and RNs)	Yes (NPs and RNs)	Yes (NPs and RNs)	Yes (NPs and RNs)	Yes (NPs and RNs) (if not residential care provider of the person)	N/A	No (but may be asked to provide information)
*End-of-Life Choices (Voluntary Assisted Dying Act) 2021* (Tas)								
South Australia (‘SA’)	No	No	Yes (NPs and RNs)	No (can provide info on request)	Yes (NPs and RNs)	Yes (NPs and RNs) (if not directly involved in providing healthcare)	Yes (NPs and RNs) (must be independent of coordinating medical practitioner)	Yes (NPs and RNs)
*Voluntary Assisted Dying Act 2021* (SA)								
Queensland	No	Yes (NPs and RNs)	Yes (NPs and RNs)	Yes (NPs)	Yes (NPs and RNs)	Yes (NPs and RNs)	Yes (NPs and RNs)	Yes (NPs and RNs)
*Voluntary Assisted Dying Act 2021* (Qld)								
New South Wales (‘NSW’)	No	Yes (NPs)	Yes (NPs and RNs)	Yes (NPs and RNs)	Yes (NPs and RNs)	Yes (NPs and RNs) (if not an employee of the coordinating or consulting practitioner)	Yes (NPs and RNs) (if not employed by the administering practitioner)	Yes (NPs and RNs)
*Voluntary Assisted Dying Act 2022* (NSW)								
Australian Capital Territory (‘ACT’)*	Yes (NPs)	Yes (RNs and NPs)	Yes (NPs and RNs)	Yes (NPs and RNs)	Yes (NPs and RNs)	Yes (NPs and RNs) (if not responsible for management of facility where person is resident)	N/A	Yes (NPs and RNs)
*Voluntary Assisted Dying Bill 2023* (ACT)								

As seen in Table 1, there is significant variation in how states and the ACT include nurses in VAD. The ACT Law will permit eligible NPs and RNs to be involved in all steps of the VAD process. In WA, Tasmania, Queensland, and NSW, nurses can act as administering practitioners but cannot assess patient eligibility.

Victoria and SA are the most restrictive states; only allowing nurses to be involved in supporting and general care, or in roles permitted to be filled by eligible ordinary members of the public (such as being a Contact Person or witnessing the patient’s written request for VAD). This means nurses in Victoria and SA cannot initiate discussions about VAD with patients or administer the substance—such actions can only be undertaken by an eligible and appropriately trained doctor.

Nurses are permitted to administer VAD medication in WA, Queensland, Tasmania, NSW, and will also be permitted to do so under the ACT Law. At the time of administration of the VAD medication, administering nurses *must* assess the patient’s voluntariness and decision-making capacity, amongst other duties (Waller et al. 2023). In WA and NSW, administering NPs must also assess that the patient’s request for VAD is enduring.

The limited inclusion of nurses in Australian VAD models creates two main issues. First, the exclusion of nurses from acting as assessing practitioners arguably contributes to access barriers for Australians seeking VAD. Evidence emerging from Victoria shows that patients and family caregivers find it difficult to locate a trained, eligible, and willing doctor to assist them in the VAD process (White et al. 2023). Yet as Hewitt et al. argue, NPs have the necessary education and skills to participate as assessing practitioners. Their exclusion to date seems to have occurred more out of an abundance of caution given the politicized nature of VAD debates or simply a failure to consider their potential role, rather than because NPs are incapable of taking such a role (Hewitt et al. 2023). Yet nurses are capable of undertaking eligibility assessments for VAD and are required to assess aspects of eligibility (such as decision-making capacity) anyway when administering a VAD substance. Hewitt et al. argue that this results in a “curious anomaly”; nurses are not currently trusted to assess these criteria during the eligibility assessment process but are required to do this at the time of administration (Hewitt et al. 2023). Allowing NPs to assess eligibility could widen the pool of practitioners who can provide VAD, thus promoting better access to VAD for Australians.

Second, limiting nurses’ formal roles to acting only as administering practitioners arguably disincentivizes them from participating at all. Limiting nurse involvement to the role of administering practitioner may prevent the development of relationships between nurses and patients and families and shows (an unjustified) distrust in nurses, according to some stakeholders (Haining, Willmott, and White 2023a, b). This limited trust in nurses to have broader involvement has been recognized as a potential barrier to nurses undertaking the role of administering practitioner (Haining, Willmott, and White 2023a, b). This is observed in WA, in which only seven out of ninety-seven trained practitioners are NPs (Voluntary Assisted Dying Board Western Australia 2023). Of the sixty-eight practitioners who have acted as administering practitioner, only one was an NP (Voluntary Assisted Dying Board Western Australia 2023).

Two Australian models have attempted to resolve these issues. Queensland has taken a unique approach by involving nurses within the governance and implementation of VAD within health services across the state, such as clinical coordinators in VAD units (Queensland Health 2022). As of 30 June 2023, of 318 authorized practitioners under Queensland’s VAD law, nineteen were NPs and 144 were RNs (Voluntary Assisted Dying Review Board Queensland, 2023). Of forty-nine administering practitioners, two were NPs and twelve were RNs (Voluntary Assisted Dying Review Board Queensland 2023). This increased nurse participation is a positive outcome for Queenslanders seeking VAD. In contrast, the ACT Law will broaden the scope of lawful providers of VAD to include NPs in the roles of coordinating practitioner and consulting practitioner. The ACT Law reflects an intentional and useful adaptation of law to meet the contextual needs of patients to increase equality in access to healthcare. It also raises questions about whether other Australian states, which will be reviewing their VAD laws over the coming years (Waller et al. 2023), may wish to follow suit.

## The Role of NPs in Canada’s MAiD Framework

Canada is currently the only jurisdiction in the world to allow NPs to assess patient eligibility for VAD (Hewitt et al. 2023). MAiD was legalized after the Supreme Court of Canada struck down the absolute prohibition on physician-assisted dying in the landmark case *Carter v Canada (Attorney General)* [2015] 1 SCR 331 (“*Carter*”). In response to *Carter,* the Canadian government amended the Canadian *Criminal Code* via Bill C-14 in 2016 to establish a legislative framework to permit MAiD. The *Criminal Code* was amended again in 2021 by Bill C-7, which made changes to eligibility criteria and procedural safeguards (Health Canada 2023). While less prescriptive than the Australian model, the Canadian *Criminal Code* provisions on MAiD require patients to be assessed as eligible by two practitioners (doctors or NPs), make a written request for MAiD, and provide consent for the administration of the medication (noting the requirement for final consent can be waived in limited circumstances).

In Canada, NPs are permitted under the *Criminal Code* to assess a person’s eligibility for access to MAiD (i.e., act as an “assessor”) and provide MAiD medication (i.e., be a “provider”). NPs constitute 5 per cent of the total number of MAiD practitioners in Canada but perform 9.4 per cent of all MAiD procedures; a percentage which has increased over time (Health Canada 2023). After family physicians, NPs provide the second highest percentage of MAiD provisions, ahead of palliative medicine specialists, internal medicine physicians, and oncologists (Health Canada 2023). In addition to formal roles as assessors or providers of MAiD, data from the most recent Canadian report on MAiD practice highlights that when health professionals involved with MAiD consult with another health professional, in 41.7 per cent of cases that consultation is with a nurse (Health Canada 2023). This evidence demonstrates that nurses are integral to the provision of MAiD in Canada in terms of their formal roles and are also a rich source of information and support beyond these formal roles.

Despite the integral role of NPs since the inception of MAiD in Canada, involvement in MAiD can be complex. Some complexities are common to both doctors’ and NPs’ experiences, such as in relation to: (1) discussions about MAiD with patients and colleagues; (2) adjusting practice in line with changing legal requirements (e.g., the waiver of final consent in some MAiD cases post Bill C-7); (3) delayed referrals, lack of care coordination, or misinformation from other providers, creating challenges in supporting patients; and (4) varying levels of institutional support (Brown et al. 2020; Pesut et al. 2021). Another key challenge in Canada is provider sustainability given the large number of patients seeking MAiD (Oczkowski et al. 2021). However, this issue of sustainability is particularly pertinent in the context of NPs, because many NPs in Canada are unremunerated for MAiD-related work. In some areas of Canada, despite MAiD being practised since 2016, there is still no remuneration model for NPs involved in MAiD and they provide MAiD care on a volunteer basis (Oczkowski et al. 2021; Pesut et al. 2021).

This challenge of remuneration resonates in the Australian context. Haining et al. identify that the Medicare Benefits Scheme (“MBS”) mechanism may be used for VAD-related consultations but that no MBS item can be claimed for the VAD administration (Haining, Willmott, Towler et al. 2023a, b), which (excluding the ACT) is currently the only formal role that NPs or RNs may undertake in the VAD process in Australia. Haining et al. conclude that providing VAD in Australia rests largely “on the goodwill of medical practitioners to undertake unpaid work” (Haining, Willmott, Towler, et al. 2023a, b), which is an unsustainable approach.

## What Next for the Role of NPs in Australian VAD Systems?

Table 1 demonstrates how NPs and RNs may be involved in VAD across Australian jurisdictions, in terms of both formal roles and more general interactions with the VAD system. The ACT Law represents a departure from the Australian model by allowing NPs to take up formal roles as coordinating practitioners and consulting practitioners. The ACT’s decision to include NPs was made in an effort to promote equal access to a legal healthcare service and may be an important step in achieving better access for Australians seeking VAD. Stakeholders have suggested that NPs ought to be trusted as assessors of eligibility (Haining, Willmott, and White 2023a, b), particularly to improve outcomes in regional areas. Evidence from Canada demonstrates that NPs have an important role to play in MAiD provision (Health Canada 2023). Though constituting a relatively small proportion of providers, they take on a large percentage of MAiD provisions, ahead of palliative medicine physicians and oncologists (Health Canada 2023). As Australian states review their VAD systems as required by each state’s enacting legislation, the time has come to consider an expanded role for nurses (Hewitt et al. 2023). This may include allowing NPs to assess patient eligibility for VAD (or at least facilitating NPs to act as administering practitioners where this is currently not allowed, such as in the restrictive models in Victoria and SA).

If the role of NPs is expanded, consideration must be given to appropriate remuneration of NPs (as well as doctors). Given that one of the key reasons for introducing a role for NPs as coordinating practitioners or consulting practitioners in the ACT rests on access, the ACT, other Australian states, and the Australian federal government must grapple with remuneration, including for NPs, to ensure this goal is achieved in practice. This is critical in ensuring workforce sustainability to meet the policy goal of increasing access by including a wider role for NPs. Experience from Canada highlights that this should be considered proactively, given that in Canada, some NPs are still practising unremunerated eight years after MAiD was legalized (Oczkowski et al. 2021; Pesut et al. 2021).

Furthermore, although nurses who participate in the VAD process as administering practitioners are required by law to undertake formal training, *all* nurses should be encouraged to undertake training in VAD and end-of-life law given their likelihood of interacting with the VAD framework within their nursing roles as supporters and general carers of patients. This could be achieved by encouraging the uptake of end-of-life law training programmes and implementation of education in medical law and ethics units within undergraduate nursing degrees.

## Conclusion

The passing of the ACT Law provides an opportunity for Australia to reconsider the scope of nurses’ roles in VAD systems. In some Australian states, NPs and RNs are able to take on formal roles in the process as administering practitioners. Nurses also have various key informal roles in the VAD process, such as providing general and supportive care to patients who may be seeking VAD or providing information to patients (acknowledging restrictions apply across states about how/when this can occur). Given evidence of patients experiencing difficulties finding doctors to assist them through the VAD process in Australia, increasing the pool of potential assessing practitioners to include NPs could help to improve access to VAD for patients. This may also relieve some of the pressures on the small VAD workforce of doctors and support provider sustainability. Canada’s MAiD system provides evidence of the important role of NPs in providing MAiD. If Australian states follow this path, evidence from Canada highlights that any future considerations must include sufficient remuneration to support nurses in this role.

## Data Availability

Not applicable.

## Footnotes

1 The ACT Law is set to commence operation on November 3, 2025, after an implementation period.
